# Spatial characterization and stratification of colorectal adenomas by deep visual proteomics

**DOI:** 10.1016/j.isci.2024.110620

**Published:** 2024-07-31

**Authors:** Sonja Kabatnik, Frederik Post, Lylia Drici, Annette Snejbjerg Bartels, Maximilian T. Strauss, Xiang Zheng, Gunvor I. Madsen, Andreas Mund, Florian A. Rosenberger, José Moreira, Matthias Mann

**Affiliations:** 1 Novo Nordisk Foundation Center for Protein Research, Faculty of Health Science, University of Copenhagen, Copenhagen, Denmark; 2 Precision Cancer Medicine Laboratory, Department of Drug Design and Pharmacology, Faculty of Health and Medical Sciences, University of Copenhagen, Copenhagen, Denmark; 3 Department of Pathology, Odense University Hospital, Odense, Denmark; 4 Department of Proteomics and Signal Transduction, Max Planck Institute of Biochemistry, Martinsried, Germany

**Keywords:** Artificial intelligence, Cancer, Cancer system biology, Proteomics

## Abstract

Colorectal adenomas (CRAs) are potential precursor lesions to adenocarcinomas, currently classified by morphological features. We aimed to establish a molecular feature-based risk allocation framework toward improved patient stratification. Deep visual proteomics (DVP) is an approach that combines image-based artificial intelligence with automated microdissection and ultra-high sensitive mass spectrometry. Here, we used DVP on formalin-fixed, paraffin-embedded (FFPE) CRA tissues from nine male patients, immunohistologically stained for caudal-type homeobox 2 (CDX2), a protein implicated in colorectal cancer, enabling the characterization of cellular heterogeneity within distinct tissue regions and across patients. DVP identified DMBT1, MARCKS, and CD99 as protein markers linked to recurrence, suggesting their potential for risk assessment. It also detected a metabolic shift to anaerobic glycolysis in cells with high CDX2 expression. Our findings underscore the potential of spatial proteomics to refine early stage detection and contribute to personalized patient management strategies and provided novel insights into metabolic reprogramming.

## Highlights

• Single-cell-type spatial proteomics characterizes colorectal adenoma heterogeneity
• Metabolic switch to anaerobic glycolysis in high-grade dysplasia regions
• DMBT1, MARCKS, and CD99 can stratify adenomas in deep visual proteomics
• Proteomics of high-grade dysplasia areas show protein signatures of recurrence

## Graphical abstract

**Figure:**
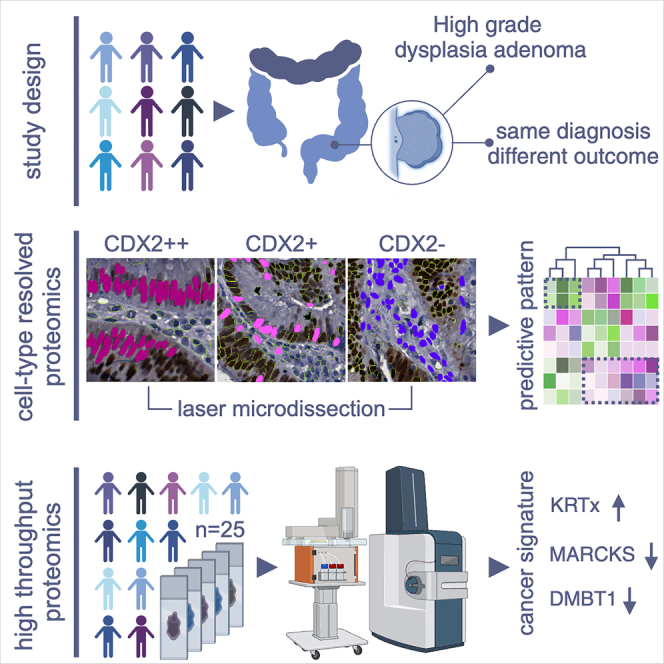

## Teaser

Artificial intelligence; Cancer; Cancer systems biology; Proteomics

## Introduction

Colorectal cancer (CRC) is one of the most prevalent cancers, with a poor prognosis when detected at later stages. Worldwide, it is the third most diagnosed cancer and the second-leading cause of cancer deaths. Two-thirds of CRC cases arise sporadically by a combination of multiple environmental risk factors, such as unhealthy diet, physical inactivity, obesity, smoking, and excessive alcohol consumption.^1^^,^^2^

CRC screening programs, together with removal of precancerous lesions—colonic adenomatous polyps (adenomas)—have led to decreasing incidence rates in older adults in high-income countries, whereas incidence is rising in developing countries, at least for adults under 50 years.^2^ Regular screenings strongly decrease CRC-associated mortality because detected adenomas are removed, followed by scheduled follow-up screening based on a set of criteria, including the number of adenomas removed, their size, and histopathologic presentation.^3^ The presence of high-grade (HG) dysplasia categorizes a precursor lesion as high-risk, requiring patients to undergo regular endoscopic examinations. Given that only 5–10% of HG dysplasia adenomas will ever develop into carcinomas, this leads to overtreatment and frequent surgical removals of nonmalignant colorectal polyps, a burden for both the individual and the healthcare system.^4^^,^^5^^,^^6^ It is therefore important to develop a more sophisticated and robust workflow for colorectal adenomas (CRA) classification that is supported by quantitative molecular data rather than only immunohistochemistry (IHC) and cell morphology.

Tissue samples collected for diagnostic purposes are routinely preserved by formalin-fixation and paraffin-embedding (FFPE) which can be archived in hospitals for decades. These fixed and embedded tissue samples exhibit remarkable stability, allowing for their extended use in clinical histopathological assessments, and offering the potential for continued research utilization many years after their initial preparation.^7^ However, different macromolecules are affected differently by storage time and conditions.^8^^,^^9^

In recent years, MS-based proteomic technologies have improved markedly in sensitivity and robustness, now routinely allowing large-scale analysis of FFPE tissues in a clinical context.^10^ This was enabled by advances in sample preparation protocols allowing highly efficient peptide recovery, robust and streamlined liquid chromatography (LC) set ups, and increasingly powerful mass spectrometry (MS) instruments.^10^^,^^11^^,^^12^

Proteomics experiments have typically employed data dependent acquisition (DDA) modes, in which the mass spectrometer picks the top N most abundant precursors in each mass scan for fragmentation. In contrast, data independent acquisition (DIA) repeatedly fragments segments of the mass range, which improves the sampling of peptides and data completeness.^13^^,^^14^ The resulting multiplexed spectra need to be deconvoluted by sophisticated software algorithms that match “spectral libraries” to the data. DDA is still often used for building these libraries. On the quadrupole-TOF (time of flight) mass analyzer employed here, specialized DIA methods called diaPASEF (parallel accumulation-serial fragmentation) have enabled high ion utilization, yielding high data completeness even for low-input samples.^12^^,^^15^

Very recently, deep visual proteomics (DVP) has made it possible to analyze collections of the same single-cell types or states^16^. DVP utilizes high-resolution image information, combines it with automated single-cell laser microdissection and ultra-high sensitivity mass spectrometry (MS) and is readily applied to FFPE material.

In this work, we set out to develop a robust and streamlined DVP pipeline that could enable a spatially resolved, in-depth molecular characterization and prognostic stratification of CRAs. We selected caudal-type homeobox transcription factor 2 (CDX2) as the guiding feature for our DVP analysis of CRAs. CDX2 is a key regulator of intestinal differentiation and homeostasis.^17^ In CRC, CDX2 functions either as a tumor-suppressor gene^18^^,^^19^^,^^20^ or as an oncogene,^21^ depending on context. In addition, CDX2 regulates immune cell infiltration in the intestine, modulating local immune responses.^22^^,^^23^ Loss or decreased expression of CDX2 is a common event in CRC, being associated with molecular features, such as CpG island methylator phenotype, and microsatellite instability^24^ and may be predictive of a more aggressive disease course.^22^^,^^24^ Gain of *CDX2* is the second-most-frequent aberration occurring in colorectal adenomas, making it a frequent event in colorectal tumorigenesis.^25^ Given that CDX2 gain can predict adenoma recurrence and that mutations in the *CDX2* gene are extremely rare in CRC,^26^ this protein is well suited to guide the discovery of progression or recurrence biomarkers using DVP.

We collected HG dysplasia adenoma tissue samples from nine individuals based on their clinical history and divided them into three groups: development of either metachronous CRC (C), metachronous HG adenoma (HDA) within five years, or no new lesions at least up to 10 years of colorectal surveillance, categorized as the group of non-metachronous neoplasms (NMN). Here, we describe the results of our CDX2-guided spatially resolved, in-depth proteomic analysis of these samples.

In conclusion, our optimized DVP workflow readily integrated into well-established clinical protocols, seamlessly aligning with existing routine pathology practices. Our study furnished comprehensive spatial proteome data at the single-cell-type level, addressing the inherent heterogeneity that is intrinsic both within individual tumors and among different patients.^27^^,^^28^ This unveiled novel perspectives on region-specific protein landscapes, shedding light on biologically significant factors and spatially localized processes that could play pivotal roles in CRA classification and clinical decision-making.

## Results

### Study design and the deep visual proteomics workflow

To investigate molecular protein signatures associated with disease progression in CRA, we employed a retrospective study design that comprised a cohort of male individuals who had undergone polypectomy procedures for CRA removal.^29^ We selected a sub-cohort of nine patients that all had HG dysplasia adenomas as verified by a pathologist using morphological criteria and IHC analysis but had heterogeneous clinical outcomes. Of the nine patients, three were diagnosed with metachronous colorectal cancer (C) within a year, three developed new lesions exhibiting HG dysplasia characteristics once again (HDA), while the remaining three patients remained free of new neoplastic lesions throughout the entire 10-year surveillance duration (NMN) (Table S1).

**Figure 1:**
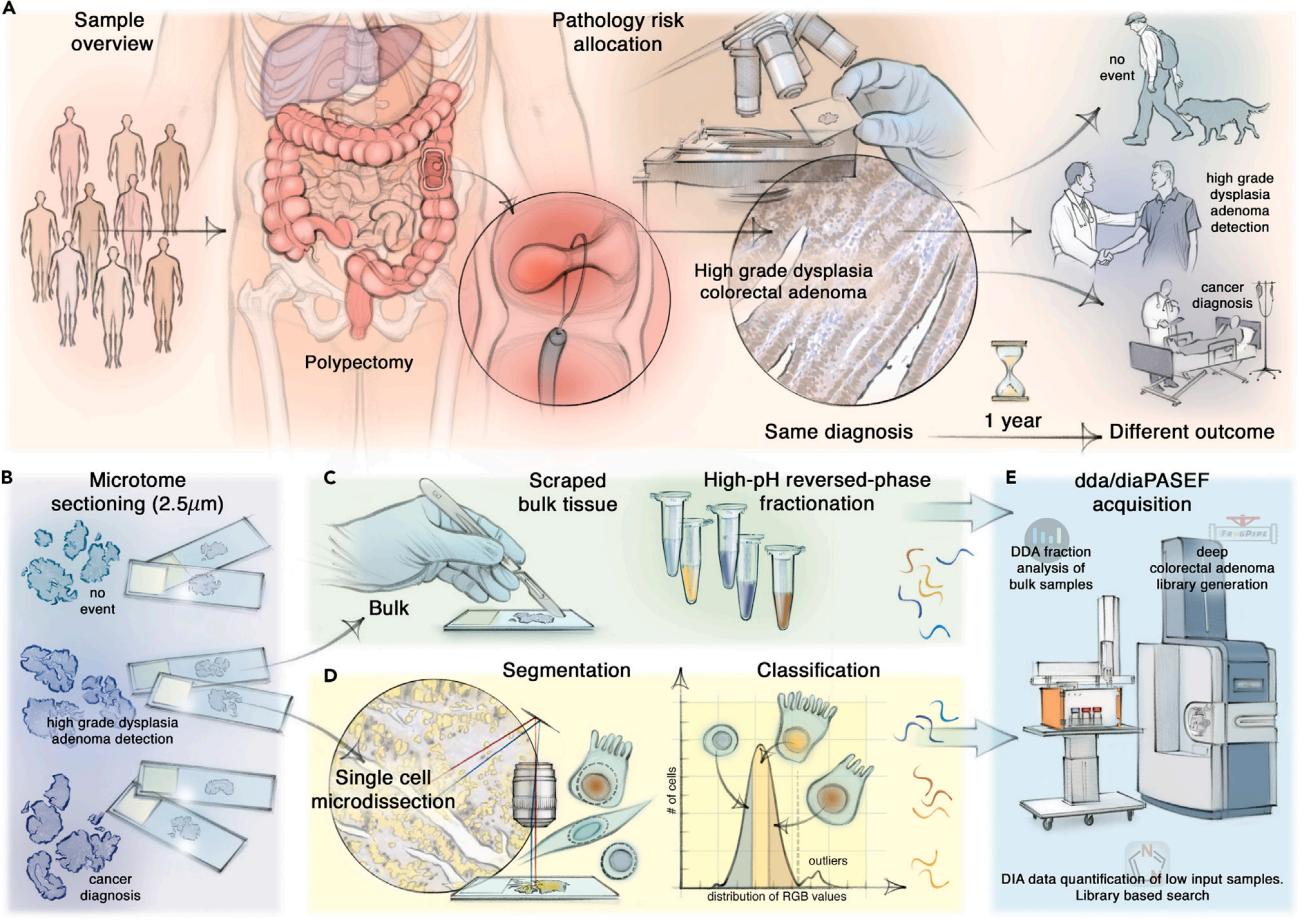
Study design and our multi-layered mass spectrometry-based proteomics approach (A) Schematic representation of the colorectal adenoma (CRA) cohort and the study design. Nine resected CRAs displayed high-grade (HG) dysplasia which led to the same pathological assessment and diagnosis but showed three different clinical outcomes. (B–E) Multi-layered and streamlined mass spectrometry (MS)-based proteomics approach applied to FFPE CRA tissues. (B) FFPE blocks of each polyp were cut and mounted onto PEN membrane slides. (C) For bulk proteomics analysis, each tissue was scraped, lysed, digested, and extensively fractionated. (D) Deep visual proteomics (DVP) workflow for the analysis of region and cell class-specific protein changes, including machine learning (ML)-based segmentation, RGB and morphology-dependent classification, followed by automated laser microdissection. (E) Bulk proteomics and low-input DVP samples were measured on the same EvoSep-timsTOF platform, either in data-dependent (ddaPASEF) or data-independent (diaPASEF) acquisition mode, followed by spectral identification and quantification with AlphaPept, MSFragger, or DIA-NN.

Note that the initial diagnosis of HG dysplasia in adenomas uniformly mandated the scheduling of costly yearly follow-up surveillance colonoscopies, a measure that, while essential for patient care, may pose practical challenges such as poor patient compliance as well as burdening the health care system (Figure 1A). This highlights the desirability of complementing existing pathology practices with molecular data.

To this end, we applied a high-throughput MS workflow which we streamlined to measure our unique cohort of nine non-malignant CRA FFPE samples, all of which were more than eleven years old. Building on standardized IHC pathology protocols and markers, we mounted each sample onto PEN membrane microscopy glass slides in duplicates and either performed bulk proteomics in DDA and DIA mode, or cell-type specific DVP (Figure 1B). For bulk analysis, we left the mounted FFPE tissues unstained and scraped them from the slides for lysis, protein digestion and subsequent extensive reverse-phase, high pH fractionation of the resulting peptides (Figure 1C, STAR Methods).

To gain insight into spatially resolved protein signatures of specific cell classes, we stained each CRA tissue for CDX2 and counterstained with hematoxylin. We combined simple IHC staining and widefield image acquisition, which we coupled to a machine learning-based nucleus segmentation model. We then classified cells based on CDX2 staining. After outlier elimination, segmented contours were categorized into three classes based on normalized RGB intensities, ratios, and form features (CDX2++, CDX2+, and CDX2-, Figure 1D). Class CDX2++ and CDX2+ were epithelial colon cells, characterized by high and medium marker expression, respectively. In contrast, the CDX2- class denotes stromal cells, identified not just by the absence of the CDX2 marker but also by differences in cell size and eccentricity.

We analyzed each class in triplicate for each patient. In each case we excised about 1,000 shapes, corresponding to about 100 complete cells in these thin sections, by automated laser microdissection, and collected those into a 384-well plate (Figure 1D). Using the EvoSep One chromatography system and the 30 samples per day method (“30 SPD”), we were able to robustly measure hundreds of FFPE-derived bulk and low-input samples on our timsTOF mass spectrometer (Figure 1E).

To obtain an in-depth proteome overview for each patient, we acquired fractions in DDA mode and quantified them using AlphaPept,^30^ our open-source Python-based MS search engine. Additionally, we employed these files to construct a very extensive CRA library by the FragPipe computational proteomics platform with integrated MSFragger.^31^^,^^32^ In contrast, all low-input DVP samples were acquired in DIA mode and searched using the DIA-NN against our comprehensive, project-specific CRA library^33^ (Figure 1E, STAR Methods).

### A very deep proteomics resource of non-malignant colorectal adenomas

**Figure 2:**
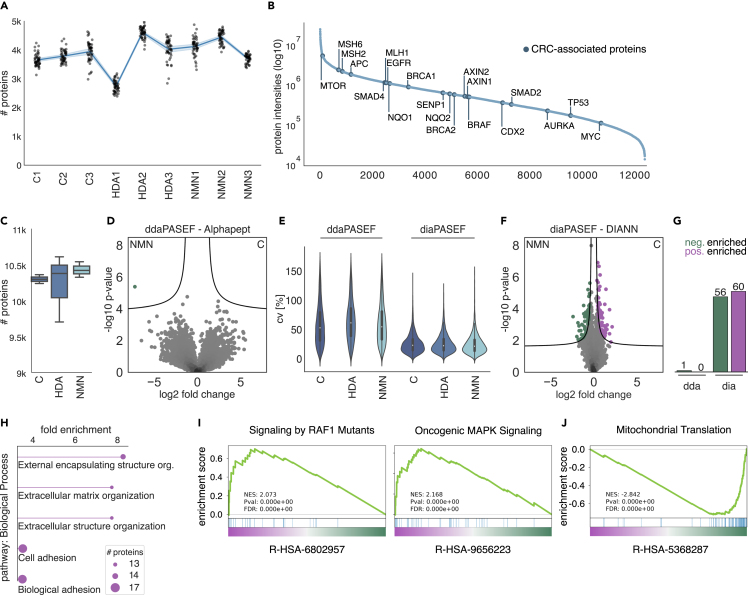
Bulk FFPE tissue proteomics of non-malignant colorectal adenomas (A) Number of proteins in each DDA-acquired fraction per CRA sample. (B) Normalized total intensity of all identified proteins in our deep CRA library created from 432 fraction samples. Highlighted in dark blue: colorectal cancer (CRC)-associated proteins, part of the TARGET (tumor alterations relevant of genomics-driven therapy) database. (C) Number of proteins per patient within a group. (D–F) (D) Pairwise proteomic comparison between C and NMN patient adenoma samples, acquired in DDA, or (F) DIA mode, and the respective (E) coefficient of variation (CV). DDA data originated from fractionated samples, DIA was measured as single run (50 ng). Significantly enriched proteins are colored and displayed above the black lines indicating statistical significance (two-sided t test, permutation-based FDR <0.05, s_0_ = 0.1). (G) Number of significantly down- and upregulated proteins in the volcano plot analyses. (H) GO biological process enrichment (FDR <0.05) of significantly upregulated protein hits. (I and J) Gene Set Enrichment Analysis (GSEA) of diaPASEF acquired data, of (I) positively and (J) negatively enriched pathways.

To obtain maximal proteomic depth from our archival, room temperature stored FFPE CRA tissues, we applied our DVP sample preparation protocol, including extensive reverse-phase, high pH fractionation, resulting in 48 fractions per patient and 432 samples in total. We measured these in just 14 days on our liquid chromatography system, coupled to our ultra-high sensitivity mass spectrometry instrument (STAR Methods). Median protein depth across samples was nearly 4,000 unique proteins, summing up to deep CRA library of 12,380 proteins from 178,274 unique, identified peptides, all at a false discovery rate (FDR) of 1% (Figure 2A). Given that there are about 20,000 protein coding genes, this constitutes excellent proteomic coverage, which was also supported by identification of 79% of TARGET (tumor alterations relevant of genomics-driven therapy) database-annotated genes known to be associated with disease progression, CRC, and resistance (Figure 2B).^34^^,^^35^

We then selected the maximum intensity value for each protein across the fractions of each patient for differential analysis, which yielded a median protein number of more than 10,000 for each of the patients (Figure 2C). We expected the largest biological variation between those that had developed cancer and those that with no neoplasms after 10 years (C and NMN), and performed a two-tailed Student’s t test with multiple-hypothesis testing correction (permutation-based FDR <0.05, s_0_ = 0.1) between them (Figure 2D). However, this analysis resulted in only minor differential expression, possibly because of the small number of patients and the relatively high coefficient of variation (CV), caused by the extensive fractionation procedure (Figure 2E).

Next, we measured unfractionated lysate for each patient directly in the DIA mode using the deep DDA library for matching. Supporting our conjecture, this resulted in many more statistically significantly regulated proteins (60 upregulated in the cancer group and 56 upregulated in the NMN group) (Figures 2F and 2G). Gene ontology (GO) term enrichment on the upregulated proteins in DIA data revealed significant effects on processes related to extracellular structure reorganization and cell-cell adhesion (Figure 2H). Moreover, Gene Set Enrichment Analysis (GSEA) identified the involvement of RAF1, oncogenic MAPK signaling and mitochondrial translation pathways (Figures 2I and 2J). This finding reflects augmented proliferation and cell migration within CRAs of group C, alongside a metabolic shift favoring anaerobic glycolysis.^36^^,^^37^ Although this proteomics analysis constitutes positive control as it largely recapitulates known features of CRC, it clearly suffers from the limitation of bulk tissue analysis, in terms of spatial resolution and in assigning these effects to specific cell types, pointing to the desirability of DVP analysis. As a preliminary step, we next analyzed down to 5 ng of FFPE bulk tissue, an amount that would readily be available in DVP (Figure S1A). Encouragingly, we found similar protein signatures in these experiments (Figure S1B).

### Characterization of the colorectal adenoma landscape by DVP

**Figure 3:**
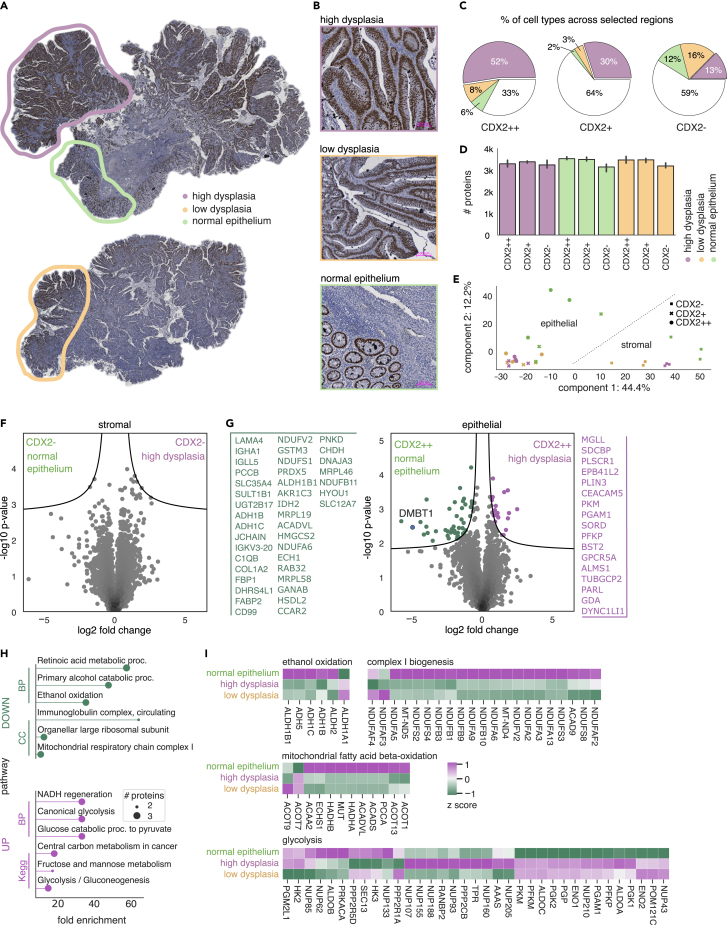
DVP characterizes region-specific metabolic changes within strongly heterogenous CRA sample (A) IHC staining of patient tissue C3 with three annotated tumor areas. (B) Representative images of selected regions based on the degree of dysplasia, density of CDX2++ epithelial cells and lymphocyte infiltration (also see Figure S3D). The color code signifies: 1, high dysplasia with a high density of CDX2++ cells (purple); 2, low dysplasia and medium density of CDX2++ cells (yellow); 3, normal glandular architecture and lymphocyte infiltration (green). Scale bar, 100 μm. (C) Distribution of CDX2++, CDX2+, and CDX2- cells across annotated regions and the remaining whole tissue area (white). Note that percentages are rounded and may not add up to 100%. (D) Unique protein numbers identified in CDX2++, CDX2+, and CDX2- cells. 1000 contours collected in triplicates. (E) Principal-component analysis (PCA) of collected CDX2++ and CDX2- cells across respective regions. (F and G) Pairwise proteomic comparison of CDX2- and CDX2++ between the highly dysplastic area and the region with normal epithelium (two-sided t test, permutation-based FDR <0.05, s_0_=0.1). (H) GO term enrichment (FDR <0.05) of positively (purple) and negatively (green) enriched proteins. (I) Cluster map of reactome-annotated pathways. Normalized and standardized intensity values were used as input.

Before applying our spatial DVP workflow across the different cell types in the CRA cohort, we first explored the protein landscape of the disease in one individual. We chose a particularly heterogeneous adenoma that was surgically removed from the colon of a male individual (designated C3 in Table S1). In the following year, this patient had been diagnosed with CRC upon surveillance follow-up colonoscopy. For this case study, we stained the tissue by IHC against CDX2 as a marker for HG dysplasia that is associated with colorectal tumorigenesis. Based on expression of this marker and tissue morphology in a widefield image, we chose three tissue areas which are characterized by: focal high dysplasia (region 1, purple); low dysplasia (region 2, yellow); as well as a region with normal glandular architecture and strong lymphocyte infiltration (region 3, green) (Figures 3A and 3B; Figures S2A and S2E). Quantifying the distribution of classified cells across the whole tissue and annotated areas, we found that 52% of all CDX2++ and 30% of CDX2+ cells were located in the high dysplasia region. The proportion of CDX2- stroma cells, however, was similar in all regions (12–16%, Figure 3C).

Next, we applied our DVP workflow to microdissect 1,000 shapes from each class in triplicate within our defined regions. The resulting protein amount is equivalent to about 100 intestinal enterocytes, and we reached a median of 3,433 unique proteins, with slightly lower numbers for smaller CDX2- stromal cells compared to larger columnar enterocytes (Figure 3D). Correlation analysis of the triplicates revealed high reproducibility of our DVP workflow (Pearson’s R > 0.92). Comparing the three cell types in the three regions in a correlation cluster matrix revealed high proteomic similarity between epithelial cells within and across areas (CDX2++ and CDX2+, Pearson’s R > 0.9). The proteomes of stromal cells (CDX2-) were less similar to the CDX2++ and CDX2+ cells (Pearson’s R < 0.87). However, they were still quite similar across regions (Figure S2B).

In a principal-component analysis (PCA), the three cell types separated along component 1. Stromal, CDX2- cells clustered together independently of the region. In contrast, epithelial, CDX positive cells were separated in component 2, representing high dysplasia regions vs. those with normal epithelium (Figure 3E). To further explore these differences between epithelial and stromal cells at the molecular level, we compared the proteomes of CDX2++ with CDX2- cells of the same region, which resulted in 1,324 significantly differentially expressed proteins (Figure S2C). Of those, positively upregulated proteins of the stroma were involved in pathways, such as “extracellular matrix organization” or “collagen binding” after GO term enrichment analysis, as expected because of the differences in cell types (Figure S2D).

Based on the correlation analysis and the PCA, there was only minor proteomic difference between CDX2++ and CDX2+ cells within and between regions. We therefore continue analysis only on the CDX2++ cells. When comparing CDX2-, stromal cells between the region with high dysplasia and the region with normal glandular architecture, we found no significantly regulated proteins (Figure 3F). In contrast, 70 proteins differed significantly between these regions for CDX2++ cells (Figure 3G). The second most downregulated protein in high dysplasia regions compared to the region with normal epithelium and immune infiltration was “deleted in malignant brain tumors 1” (DMBT1), a known tumor suppressor involved in mucosal immune defense.^38^

In a GO term enrichment analysis, the proteins that exhibited the most significant decrease in the high dysplasia region were primarily indicative of a transition toward increased metabolic activity of the glycolytic pathway (Figure 3H). In contrast, we observed a decline of proteins involved in the “respiratory electron transport” and a significant increase of proteins like PFKP, PKM, and ALDOC that promote anaerobic glycolysis (Figure 3H). When we filtered the proteins for the reactome-annotated enriched pathways based on identified GO terms and performed hierarchical clustering of normalized and standardized intensities, we found additional members of the aldehyde (ALDH) and alcohol (ADH) dehydrogenase family to be of consistently lower abundance in regions with high dysplasia. The only exception was ALDH1A1 where protein abundance positively correlated with the severeness of dysplasia (Figure 3I). Further, examining “complex I biogenesis” proteins, it became evident that nearly all of the detected NADH-uniquinone oxidoreductase (NDUF) family members were less abundant in areas of high and low dysplasia (Figure 3I). These proteins are essential integral components of the NADH-quinone oxidoreductase in the mitochondrial oxidative phosphorylation system, thus DVP directly and *in situ* captured the metabolic change from oxidative phosphorylation toward anaerobic glycolysis specifically in CDX2++ epithelial cells of high dysplasia regions, also termed “Warburg effect”.^39^

### Protein levels of DMBT1, MARCKS, and CD99 stratify the CRA cohort

**Figure 4:**
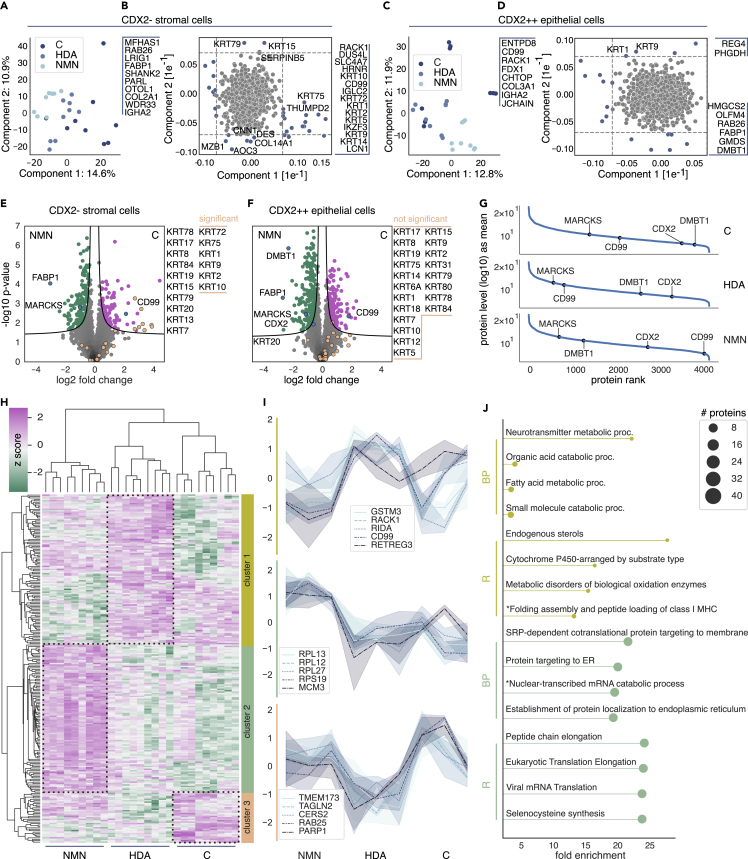
Singly isolated CDX2- and CDX2++ cells from colorectal adenoma tissues from group C, HDA, and NMN reveal a potential biomarker set for patient stratification (A–D) Principal component analysis (PCA) of collected (A and B) CDX2-stromal and (C and D) CDX2++ epithelial cells across all nine colorectal adenoma tissues. (E and F) Pairwise proteomic comparison of (E) CDX2- and (F) CDX2++ cells comparing the cancer group to NMN (two-sided t test, FDR <0.01, s_0_ = 0.1). (G) Ranked protein abundance of normalized mean intensities of all identified proteins within each CRA group. The potential markers for adenoma classification DMBT1, CD99, and MARCKS are highlighted and labeled. (H) Unsupervised hierarchical clustering of 244 ANOVA significant proteins (permutation-based FDR <0.01, s_0_ = 0.1). (I) Line graphs of the top five proteins with the highest ANOVA q value per cluster. (J) GO term enrichment analysis of cluster 1 and 2 (FDR <0.05), highlighting biological process (BP) and reactome (R) pathways of proteins with a positive *Z* score.

To investigate whether we can stratify our colorectal adenoma tissues into recurrence groups C, HDA, and NMN, we automatically microdissected 1,000 shapes of CDX2- stromal and CDX2++ epithelial cells from HG dysplasia areas of all nine samples, from which we quantified a median of more than 4,200 unique proteins per sample. The PCA analysis clearly demonstrated a gradual separation among the groups C, HDA, and NMN in both cell-types enriched samples (Figures 4A and 4C), with different proteins driving the proteomic clustering.

In CDX2- cells, the separation of groups was greatly influenced by keratins (Figure 4B), whereas in CDX2++ epithelial cells, this included cluster of differentiation 99 (CD99), fatty acid-binding protein 1 (FABP1), and DMBT1 (Figures 4B and 4D). A pairwise proteomic comparison of CDX2- cells between group C and NMN underscored keratins and CD99 as significantly enriched proteins in group C (Figure 4E), whereas CDX2 was not detected. Myristoylated alanine-rich protein kinase C substrate (MARCKS) and FABP1 were both predominantly enriched in the NMN group (Figure 4E). This pattern was consistent when comparing CDX2++ cells, with CD99, MARCKS, and FABP1 significantly enriched; with no differential expression of keratins and CDX2 as expected. Remarkably, the protein DMBT1 appears to be the strongest outlier (fold change and significance) that was downregulated in the C group—a pattern that is exclusive to CDX2++ epithelial cells (Figures 4F and Data S1).

Given our observation that CDX2++ cells most directly reflect the progression from adenoma to cancer, we evaluated the relative abundance of candidate proteins MARCKS, CD99, and DMBT1. DMBT1 showed the strongest downregulation in the C group, increasing in abundance in HDA and NMN and in a more indicative manner than the established marker CDX2 (Figure 4G). Conversely, the levels of CD99 were high in groups C and HDA and decreased substantially in NMN (Figure 4G). MARCKS displayed a moderate decrease in abundance from C to NMN, which was still statistically significant (Figure 4G). These observations in the rank order plots were mirrored in the assessment of absolute log2-transformed label-free quantification (LFQ) intensities (Figure S3E).

To further explore characteristic protein patterns capable of classifying and stratifying colorectal adenomas, we performed an unsupervised hierarchical clustering (permutation-based FDR <0.01) of 244 ANOVA significant proteins hits (Figures 4H and Data S1).

Triplicates as well as outcome groups grouped together, suggesting robust proteomic difference between tumor tissues which were originally combined into the same one “high dysplasia” group. The heatmap indicated a main cluster in each of the groups, whose constituent proteins are shown in Figure 4I. Receptor for activated C kinase 1 (RACK1, UniProt ID: D6RF23) and CD99 show were among the top five most regulated in cancer and HDA samples (top profile in Figure 4J). Taken together, the proteins in this cluster were predominant in pathways for fatty acid and sterol metabolism (Figure 4J). In the middle profile, ribosomal proteins stood out as markedly up in the NMN subset (Figure 4I). Pathway enrichment analysis further highlighted “protein targeting to the endoplasmic reticulum”, “establishment of protein localization to the endoplasmic reticulum”, and “peptide chain elongation” (Figure 4I). The bottom profile exclusively corresponds to proteins up in the cancer group, with notable colon cancer proteins such as TMEM173 (or STING, stimulator of interferon genes),^40^ PARP1 (poly ADP-ribose polymerase-1),^41^^,^^42^ and RAB25 (Ras-related protein Rab-25).^43^

Taken together, applying DVP and cell-type enrichment has enabled us to identify unique protein patterns specific to CDX2- stromal and CDX2++ epithelial cells. These patterns effectively characterize and stratify adenomas, initially grouped as having similar degrees of disease aggressiveness, into distinct groups based on actual disease recurrence. Specifically focusing on CDX2++ epithelial cells within high dysplasia regions, we suggest MARCKS, CD99, and DMBT1 as potential indicators for the transition from adenoma to carcinoma. However, given our relatively small n-numbers, this would have to be verified in larger and independent cohorts.

### DMBT1 stratifies colorectal adenomas independently of cell-type enrichment

To determine if protein patterns specific to CDX2- stromal and CDX2++ epithelial cells could be replicated using alternative methods, we orthogonally investigated DMBT1, CD99, and MARCKS as potential markers for adenoma stratification.

**Figure 5:**
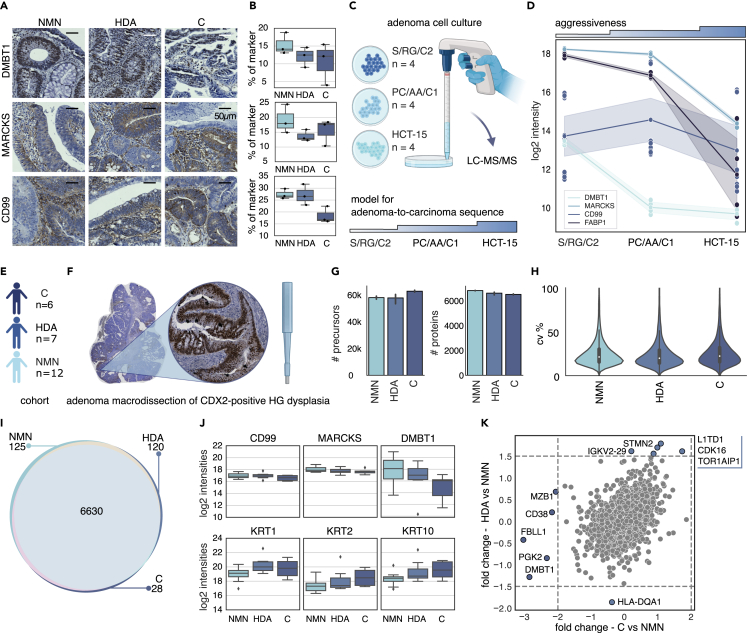
Orthogonal assessment of DMBT1, MARCKS, and CD99 in a cell culture model and an extended colorectal adenoma cohort (A) Representative CRA images of IHC staining. Scale bar, 50 μm. (B) Signal quantification of DMBT1, MARCKS, and CD99 on IHC-stained images. (C) Schematic outline of the adenoma cell culture setup and the associated aggressiveness. (D) Log2 intensity values of these marker proteins across adenoma cell lines S/RG/C2 and PC/AA/C1, and colon carcinoma cell line HCT-15. (E and F) Study design of the extended CRA validation cohort and the tissue macrodissection of CDX2-positive HG dysplasia areas. (G) Number of precursors and proteins from macrodissected CRA tissue. (H) Coefficient of variation of each CRA group. (I) Overlap of proteins between each CRA group. (J) Log2 intensity values for CD99, MARCKS, DMBT1, and keratins 1, 2, and 10 from macrodissected, CDX2-positive HG dysplasia areas in the extended CRA validation cohort. (K) Correlation of fold change between C and NMN, to HDA and NMN, after a two-sided t test, FDR <0.01, s_0_ = 0.1.

Initially, we conducted IHC staining on all nine adenoma samples. The expression patterns of DMBT1 and MARCKS aligned with our proteomic data (Figures 5A and 5B), whereas CD99 did not, likely due to its variable presence across stromal and epithelial areas (Figures 5A and 5B).

We then assessed these proteins in two established adenoma cell lines, S/RG/C2^44^ and PC/AA/C1,^45^^,^^46^ and in the colorectal carcinoma model HCT-15^47^ to mimic increased tumor aggressiveness (Figure 5C). The expression levels of DMBT1, CD99, and MARCKS correlated with the malignancy status, consistent with our previous findings (Figure 5D).

To further investigate the utility of our potential markers for pathology risk assessment, we expanded our study to include a total of 25 colorectal adenoma samples (Figures 5E; Table S2). Unlike our earlier approach using DVP for cell-type enrichment, a pathologist assessed all tissues for HG dysplasia and CDX2+ regions, which were then macrodissected using a biopsy puncher (STAR Methods, Figure 5F). This entailed the examination of a much larger area than the initial 1,000 shapes in addition to a diverse population of cell types. MS-based proteomics quantified a median of over 60,000 precursors and 6,500 proteins, for a total of 7,366 proteins with CVs around 20% (Figure 5G). Across the C, HDA, and NMN groups, 6,630 proteins were commonly quantified, with only a few proteins unique to each group (Figure 5I). Analysis of the log2 intensities of our initial candidate markers showed that CD99 mirrored the pattern observed in the IHC staining. MARCKS exhibited only a minor decrease along the NMN-HDA-C axis, whereas DMBT1 had a trend of reduction in log2 signal intensity in the HDA and C groups compared to the NMN group (Figure 5J). In addition to our proposed markers, KRT1, KRT2, and KRT10 were enriched in the C group among the stromal CDX2- population in our previous DVP experiment, consistent with the intensity increase observed in our larger adenoma cohort (Figure 5J). We further correlated the fold changes between the groups, confirming DMBT1 as the primary protein decreasing in abundance toward group C confirming the gradual decrease of DMBT1 along the NNM-HAD-C axis (Figure 5K).

## Discussion

In this paper, we applied the power of deep visual proteomics (DVP) to the important challenge of adenoma stratification beyond current clinical practice. Our goal was to develop and implement a technological framework compatible with routine histological assessments of colorectal adenomas while adding a streamlined, cell-type-specific proteomics readout. For our chosen sub-cohort of nine male patients representing three outcomes (cancer, high-dysplasia adenoma, and non-metachronous neoplasms after ten years), we first generated a deep spectral library by DDA measurements of extensively fractionated bulk samples of each of these samples, leading to an in-depth CRA proteome, a valuable resource of more than 12,000 unique proteins. We found minimal significant differences between the outcome groups due to insufficient quantitative accuracy in these fractionated, DDA data. This was partially alleviated by bulk DIA measurements that used the deep spectral libraries acquired by DDA. However, to move beyond global features, such as enrichment of proteins involved in extracellular matrix (ECM) organization or cell adhesion in C, suggesting structural changes which are in line with enhanced tumor cell migration and invasion,^48^^,^^49^ we needed to move to a cell-type-specific approach.

We then turned to deep visual proteomics to investigate intra-tumor heterogeneity. Starting with one particularly heterogeneous adenoma sample, we defined regions with increasing levels of dysplasia. We further defined three cell types based on the staining of CDX2, a common marker for HG dysplasia, namely CDX2++, CDX2+, and CDX2-. This immediately revealed differences in energy metabolism across different regions, which were attributable to CDX2++ epithelial cells. These notably increased proteins associated with glycolysis and decreased mitochondrial complex I proteins in regions with high dysplasia,^50^ a direct, *in situ* and cell-type-specific observation of the Warburg effect. DVP assigned this change specifically to highly dysplastic areas within the tumor, rather than being observed throughout the entire adenoma polyp. In addition, we observed a reduced expression of detoxification enzymes in high dysplasia areas which may cause intensified carcinogenesis.^51^ Specifically, ALDH1A1 levels increased in CDX2++ cells in regions of high dysplasia compared to other members of the aldehyde dehydrogenase protein family. This protein primarily participates in the oxidation process of retinaldehyde to retinoic acid, which promotes cell proliferation and inhibits apoptosis through the action of the transcription factor and proto-oncogene c-MYC.^52^^,^^53^ Differential expression of ALDH and AHD family members has been controversially discussed as either promoting or inhibiting cancer progression. Our data support that ALDH1A1, which has already been proposed as a prognostic maker for early invasiveness of cancer,^54^^,^^55^ is clearly upregulated in regions of high dysplasia.

Given the detailed insights into tumor heterogeneity provided by cell-type enrichment using DVP, we expanded our proteomics investigation to include CDX2- stromal and CDX2++ epithelial cells across all nine CRA individuals.

CDX2- and CDX2++ proteomes both clustered by replicates of the same patient and importantly also by outcome, suggesting the existence of protein signatures associated with the adenoma to carcinoma transition. Keratins, a family of structural proteins increasingly recognized as diagnostic markers in cancer progression,^56^^,^^57^^,^^58^^,^^59^ were the primary driver proteins that stratified samples based on CDX2- stromal cells.

Focusing particularly on CDX2++ epithelial cells, DMBT1, MARCKS, and CD99 were linked with different recurrence outcomes. Notably, DMBT1, known as a tumor suppressor in several cancers,^60^^,^^61^ exhibited the strongest negative fold change.^60^^,^^61^ DMBT1 expression has primarily been observed in cells of the immune system and epithelial linings,^61^^,^^62^ driving not only differentiation but also carcinogenesis when absent, which makes it promising candidate for driving adenoma-carcinoma progression. MARCKS was another interesting protein candidate as it was solely significantly enriched in the cancer group. It is a major target of PKC and has repeatedly been implicated in tumor progression.^63^ As a plasma membrane-tethered protein that shuttles into the cytosol upon phosphorylation, MARCKS functions as a regulator of cellular signaling, affecting sensitivity to programmed cell death and cell adhesion through various pathways.^63^ Although the role of MARCKS in the development of cancers remains a topic of debate, it may suppress cell growth in colorectal cancer.^63^^,^^64^^,^^65^ Like DMBT1, spatial proteomics implicates MARCKS as being involved in the transition toward cancer. Lastly, CD99 is discussed as an onco-suppressor or an oncogene.^66^ It is involved in a diverse range of molecular processes, including the reduction of miR34a/Notch/NF-κB and MAPK pathways.^67^^,^^68^^,^^69^ In our study, CD99 exhibited significant upregulation in HG dysplasia and cancer outcome groups while it was undetectable in the NMN group, suggesting it as a driver for cell migration and tumor invasiveness.^67^^,^^68^

Through various independent methods, DMBT1 emerged as a candidate that effectively stratified colorectal adenomas along the adenoma-to-carcinoma axis, irrespective of cell-type enrichment via DVP.

In conclusion, our findings offer insights into the intra- and inter-individual biology of CRAs that later manifest different outcomes. Unlike most research in colorectal cancer, our study focuses on the precursor lesions. The samples of our cohort have the advantage of having been stored for many years, allowing us to gain insights into disease recurrence. We combine imaging data with deep proteomic analysis, offering an in-depth view of the spatial protein landscape of CRAs. Our integrative approach, supplements previous research, which often focused solely on genomics, imaging, or proteomics, by revealing cell types and regions that provide insights into disease progression. We identified a subset of potential markers after cell-type enrichment that can stratify colorectal adenomas, helping to predict whether a patient may develop cancer. This study lays a foundation for future research with larger and more diverse cohorts, providing protein candidates for further validation.

By bridging a critical gap between basic cancer research and clinical application, these advances emphasize the interdisciplinary utility of spatial proteomics. The potential markers identified here could improve patient outcomes and reduce the burden on healthcare systems, especially as more young people develop colorectal cancer and require colonoscopies.

### Limitations of the study

Although, DVP allowed us to enrich for specific cell types in a spatial manner, resulting in protein signatures that stratify adenomas and enable risk assessment for a specific cell population, one of the limitations is the current study is its relatively small size. Therefore, further validation with a larger and more diverse clinical cohort is needed to strengthen these findings at both single cell-type and bulk proteomics levels. Furthermore, we found that it is challenging to directly transfer cell-type-specific DVP data to image or bulk proteomics data. This manifested in our validation experiments where macrodissection of HG dysplasia areas where bulk proteomics successfully validated DMBT1 as a stratification marker, but MARCKS and CD99 showed minimal changes since we did not specifically enrich for cell type.

## STAR★Methods

### Key resources table

**Table:** 

REAGENT or RESOURCE	SOURCE	IDENTIFIER
**Antibodies**		

Anti-CDX2	Cell Marque	Cat# 235R-16; RRID:AB_1516801
Anti-DMBT1	Atlas Antibodies	Cat# HPA040778; RRID:AB_2677132
Anti-MARCKS	Cell Signaling Technology	Cat# 5607; RRID:AB_10547885
Anti-CD99	Cell Marque	Cat# 199R-14; RRID:AB_1516787
Anti-rabbit	Thermo Fisher Scientific	Cat# A-11036; RRID:AB_10563566
Anti-Human CD3	Agilent	Cat# A0452; RRID:AB_2335677
Anti-CD20	Thermo Fisher Scientific	Cat# 50-0202-80; RRID:AB_11151691

**Biological samples**		

Colorectal adenoma FFPE tissues	Odense University Hospital, Denmark.	N/A

**Deposited data**		

Proteomics raw data	ProteomeXchange Consortium; PRIDE	PXD046999
Image raw data	Zenodo	Colorectal Adenoma IHC (CDX2) Raw Files
Code for nuclei classification	GitHub	DVP-on-Colorectal-Adenomas

**Experimental models: Cell lines**		

Colorectal adenoma S/RG/C2	Butt et al., 1997^44^	RRID:CVCL_IQ11
Colorectal adenoma PC/AA/C1	Williams et al., 1990,^46^ Williams et al 1991^45^	RRID:CVCL_IQ04
Adenocarcinoma HCT-15	Gonçalves et al., 2022^47^	RRID:CVCL_0292

**Software and algorithms**		

AlphaPept, v0.4.1	Strauss et al., 2021^30^	N/A
MSFragger, v18.0	Kong et al., 2017^32^	N/A
IonQuant, v1.8.9	Kong et al., 2017^32^	N/A
Philosopher, v4.2.2	da Veiga Leprevost F, Haynes SE, Avtonomov DM et al., 2020^70^	N/A
EasyPQP, v0.1.25	https://github.com/grosenberger/easypqp	N/A
DIA-NN, v1.8.1	Demichev et al., 2020,^33^ Demichev et al., 2022^71^	N/A
Perseus, v2.0.5.0	Tyanova et al., 2016^72^	N/A
Python, v3.9.7	https://github.com/python/	N/A
NumPy, v1.20.3	https://github.com/numpy	N/A
Pandas, v1.3.4	https://github.com/pandas-dev	N/A
Matplotlib, v3.4.3	https://github.com/matplotlib	N/A
Seaborn, v0.12.2	https://github.com/seaborn	N/A
BioRender	https://www.biorender.com/	N/A

### Resource availablity

#### Lead contact

Further information and requests for resources and reagents should be directed to and will be fulfilled by the Lead Contact, Matthias Mann (mmann@biochem.mpg.de).

#### Materials availability statement

This study did not generate new unique reagents.

#### Data and code availability

• The proteomics raw data have been submitted to the ProteomeXchange Consortium through the PRIDE partner repository (https://www.ebi.ac.uk/pride/) with the identifier PXD046999. Image raw data have been made publicly accessible on the Zenodo platform, housed under the repository titled ‘Colorectal Adenoma IHC (CDX2) Raw Files’.
• The code for classifying segmented nuclei in colorectal adenoma FFPE tissues we deposited on GitHub, in the ‘DVP-on-Colorectal-Adenomas’.
• Any additional information required to reanalyze the data reported in this paper is available from the lead contact upon request.

### Experimental model and study particpant details

#### Human subjects

The experimental design including the Deep Visual Proteomics workflow was approved by the National Committee on Health Research Ethics (j.nr. 2112779), and a waiver for obtaining informed consent was granted (as per section 10; subsection 1 of the Committee Act).

We included a total of 25 individuals in this study, all of whom are male. For some tissues, the information about the individual’s sex is unavailable. Race, ethnicity and socioeconomic status was not documented by the pathology department. Patient demographics and clinical characteristics are shown in Tables S1 and S2.

#### Cell lines

The human colorectal adenoma cell lines PC/AA/C1 and S/RG/C2 were kindly provided by Dr. Ann Caroline Williams (Colorectal Tumor Biology Group, University of Bristol). Both lines were cultured in Dulbecco’s modified Eagle’s medium (DMEM) (Thermo Fisher Scientific, MA, USA) containing 20% fetal bovine serum, and supplemented with 1 μg/mL hydrocortisone sodium succinate, 0.2 U/mL insulin (Sigma-Aldrich; Merck, MO, USA), 2 mM glutamine, and 100 U/mL penicillin and 100 μg/mL streptomycin (Thermo Fisher Scientific).

### Method details

#### Study design and sample collection

This is a retrospective study, based on adenomas resected between 2006 and 2013 at Odense University Hospital and formalin-fixed and paraffin-embedded for preservation. Samples were provided by the Danish pathology databank (Patobank) and anonymized, after reevaluation of high-grade dysplasia status of each poly by a pathologist. Using clinical data on disease recurrence, we categorized nine CRAs into three groups of three: group C (cancer), HDA (high-grade dysplasia adenoma), and NMN (non-metachronous neoplasms) to perform our spatial proteomics pipeline Deep Visual Proteomics (DVP) on. To validate predictive protein patterns we enlarged our cohort to 25 colorectal adenoma polyps.

#### Immunofluorescence staining

The tissue sections underwent deparaffinization and hydration through three cycles of xylene and decreasing ethanol concentrations from 99.6% to 70%. Antigen retrieval was achieved by immersing sections in 10 mM citrate buffer (pH 6.0) at 90°C for 20 min. Subsequently, tissues were blocked with 5% BSA for 1 h at room temperature. Following overnight incubation at 4°C with anti-CDX2 (235R-15, Cell Marque; 1:1000) antibodies, slides were washed and then incubated with Alexa Fluor 568 goat anti-rabbit antibody (A-11036, Invitrogen; 1:1000) for 1 h at room temperature. After rinsing, slides were further incubated with anti-CD3 antibody (A0452, Agilent; conjugated with DyLight 488 (ab201799, Abcam); 1:300) and anti-CD20 (50-0202-80, Invitrogen; 1:100) overnight at 4°C. DAPI was used for counterstaining, and slides were mounted with Thermo Fisher Diamond Antifade mounting media before examination under an AxioScan7 microscope.

#### Immunohistochemistry

A detailed protocol for FFPE tissue mounting and staining on membrane PEN slides 1.0 (Zeiss, 415190-9041-000) is provided in our original DVP article.^16^

The antibodies we have used to either characterize HG dysplasia in CRAs, or for marker validation were always counterstained with Mayer’s hematoxylin. Each tissue was incubated overnight at 4°C with one of the following antibodies: anti-CDX2 (Cell Marque, EPR2764Y; 1:2000), anti-DMBT1 (Merck, HPA040778; 1:100), anti-MARCKS (BioNordika, #5607; 1:1000), or anti-CD99 (Cell Marque, 199R-14; 1:3000).

#### Laser microdissection

After reference point alignment at the LMD7 (Leica) microscope, shape contours were imported for semi-automated laser microdissection at the following setting: power 32, aperture 1, speed 20, final pulse −1, head current 42%, pulse frequency 2,600 and offset 180/220. All experiments were controlled with the LMD software v8. 1000 shapes were cut and sorted into 384-well plates (Eppendorf, 0030129547), avoiding the collection in the outermost rows and columns. After microdissection, plates were sealed, centrifugated at 1,000g for 10 min and then frozen at −20°C until further processing.

#### Macrodissection with a biopsy puncher

To facilitate the fast and straightforward macrodissection of areas with high-grade dysplastic adenomas, we used a biopsy puncher with a diameter of 1.5 mm (Miltex, 69031-02) on tissue sections mounted on 1.0 mm membrane PEN slides (Zeiss, 415190-9041-000). This approach enabled the sampling of tissue areas measuring approximately 1.7 million μm^2^. Owing to the visibility of the excised tissue to the naked eye, these small formalin-fixed paraffin-embedded (FFPE) tissue fragments were then carefully transferred using forceps into a 384-well plate.

#### High-resolution microscopy

IHC-stained FFPE tissue sections of 2.5 μm thickness were scanned with the Zeiss Axio ScanZ.1 microscope. With an ×20, 0.8 NA dry objective, widefield images were acquired using a VIS LED light source and captured by a CCD Hitachi HV-F202CLS camera. Dependent on given tissue irregularities on PEN membrane slides, the z stack configurations were set to five to fifteen slices and a regular interval of 1.50 μm to guarantee sample coverage and optimal focus. Having ‘EDF active’ (Extended depth of focus) checked during acquisition, a 2D-projection based on maximum intensity values was created and used to generate a stitched tissue image (Zeiss ZEN 2.6, blue edition) for further image processing.

The raw image files have been made publicly accessible on the Zenodo platform, housed under the repository titled ‘Colorectal Adenoma IHC (CDX2) Raw Files’.

#### Cell segmentation and classification

The DVP approach in this investigation focuses on standardized IHC and H&E staining, where cellular borders are often indistinct. We therefore utilized a deep neural network in BIAS for cell segmentation that was trained on a 'generic nuclei dataset' and set a fixed cutting-offset of 2 μm for including the cytoplasm in proteome analysis. To ensure satisfactory model performance for our purposes, we visually inspected a selection of representative image tiles. This confirmed that the model accurately delineated the nuclei in our immunohistochemically stained tissue sections. To classify epithelial CDX2 cells, we exported the image feature matrix from BIAS to a custom Jupyter Notebook. After removing outliers via a 5% z-scored intensity cutoff, cells were classified into four groups using nuclei RGB intensities and morphologies such as size and eccentricity. The processed matrix was returned to BIAS for contour export and laser microdissection. See Figure S4 for a graphical illustration of the DVP workflow, along with the specific criteria used for classification.

The code for the classification of segmented nuclei in colorectal adenoma FFPE tissues is publicly available on GitHub in a repository named ‘DVP-on-Colorectal-Adenomas’.

#### MS sample preparation

One thousand dilated nuclei contours, equivalent to about 100 colonic epithelial cells (BNID 111216), were automatically excised with the cutting offset and pooled into a 384-well plate (Eppendorf, 0030129547). To concentrate the cell shapes, 28 μL of 100% acetonitrile was added to each well, followed by centrifugation at 2,000 g for 10 min and vacuum evaporation at 60°C for 15 min. Cell lysis was achieved by adding 4 μL of 60 mM triethylammonium bicarbonate (TEAB) in water to each well and heating at 95°C for 60 min. Proteins were de-crosslinked by adding 1 μL of 60% acetonitrile to achieve a final concentration of 12% (v/v), followed by incubation at 75°C for 60 min. Subsequently, proteins were then digested in two steps: first, with 1 μL of 4 ng/μL LysC for 3 h, and then with 1.5 μL of 4 ng/μL trypsin overnight at 37°C. Enzymatic digestion was stopped by adding 1% (v/v) trifluoroacetic acid. The samples were then centrifuged for 5 min at 1,000 g and vacuum dried at 60°C. Finally, samples were vacuum dried and stored at −20°C, or resuspended in 20 μL Evosep buffer A (0.1% formic acid v/v) for direct Evotip pure loading (www.evosep.com). All MS sample preparation steps and buffers were replicated from the original DVP paper, but semi-automated by using the Agilent Bravo liquid handling robot. The sample preparation was performed using an Agilent Bravo automated liquid handling platform.

#### High-pH reverse-phase fractionation

We used high-pH reverse-phase fractionation to create a deep spectral library of CRA material for subsequent used in Data Independent Acquisition (DIA). To this end, we utilized our automated Opentrons platform for fraction collection coupled to a nanoflow HPLC (EASY-nLC 1000 system, Thermo Fisher Scientific). For the bulk analysis, FFPE adenoma tissues were scraped from the PEN glass slide and enzymatically digested for MS analysis, using the same protocol as for the DVP sample preparation (see above). Peptides were then separated on an analytical column (250 μm × 30 cm, 1.9 μm, PepSep, Bruker Daltonics) by a 100 min gradient with an exit-valve switch every 30 s and concatenated into 48 fractions.

#### LC-MS

To ensure minimal loss of peptides, we directly queued our samples after Evotip loading for LC-MS analysis. Acquisition was performed on a timsTOF instrument (Bruker Daltonics, timsTOF SCP) coupled with a Evosep One system. Using the 30 Sample Per Day (SPD) method (www.evosep.com), samples were separated on an analytical column (150 μm × 15 cm, 1.5 μm; PepSep, Bruker Daltonics), and a 10 μm emitter operated inside a nano-electrospray ion source (CaptiveSpray, Bruker Daltonics). Samples were either measured in data-dependent (ddaPASEF) or data-independent (diaPASEF) modes. For both scan modes, the ion accumulation and elution time in the TIMS tunnel was set to 100 ms. The ion mobility range was defined from 1/K0 = 1.6 Vs. cm^−2^ to 0.6 Vs. cm^−2^ and the total m/z range from 100 to 1,700. For ddaPASEF, one full cycle consisted of one MS1 survey scan followed by 10 MS/MS scans (PASEF scans). Precursor ions for MS/MS analysis were isolated using a 2 Th window for m/z values less than 700 and a 3 Th window for m/z values greater than 700. Singly charged precursor ions were excluded based on their position in the m/z and ion mobility space using a polygon filter. Precursors for MS/MS were selected at an intensity threshold of 1,500 arbitrary units (a.u.) and re-sequenced until a target value of 20,000 a.u. was reached. To avoid re-sampling, a dynamic exclusion period of 40 s was defined. For diaPASEF, we used a standard method that covers an m/z-range from 400 to 1200 Da ensuring comprehensive coverage and accurate analysis.^12^^,^^73^ The method consists of 16 diaPASEF scans, each subdivided into 4 ion mobility windows with an isolation width of 25 Th. This resulted in a total cycle time of 1.81s. Quality control samples and our deep CRA library were measured in ddaPASEF. DiaPASEF was used for low input FFPE DVP samples.

### Quantification and statistical analysis

#### MS data analysis

Bruker timsTOF ddaPASEF raw files were analyzed with AlphaPept (version 0.4.1)^30^ using standard settings (https://mannlabs.github.io/alphapept/settings.html). For our 432 deep CRA samples, each of the 48 fractions was assigned to a patient in AlphaPept. Using the same DDA files, we created a project-specific CRA library in MSFragger (v18.0) with 178,274 precursors and 12,389 unique protein groups, excluding cysteine carbamidomethylation as fixed modification.^32^ Its IonQuant (v1.8.9) and Philosopher (v4.2.2) modules handled quantification and False Discovery Rate (FDR) correction, respectively. Low input DVP samples in diaPASEF mode were analyzed in DIA-NN (v1.8.1),^33^^,^^71^ using a library-based approach against the UniProt database with isoforms (2019, UP000005640_9606). Settings included trypsin specificity with one missed cleavage, 1% precursor FDR, 15 ppm accuracy, and enabled ‘match between runs’. N-terminal methionine excision, methionine oxidation and N-terminal acetylation were left checked, and maximal 2 variable modification were allowed.

#### Spectral library generation

For low input DVP sample analysis which were acquired in diaPASEF, we utilized our project-specific deep colorectal adenoma library created using FragPipe^33^ (version 17.1, incorporating MSFragger 3.4,^31^^,^^32^ Philosopher 4.1.1,^70^ Python 3.9.7, and EasyPQP 0.1.25, available at https://github.com/grosenberger/easypqp). While default parameters were largely maintained, adjustments were made to set the precursor mass tolerance between −20 and 20 ppm and the fragment mass tolerance at 20 ppm. The resulting data tables were subjected to a 1% false discovery rate (FDR) filter using Percolator and ProteinProphet options in FragPipe.

#### Bioinformatic analysis

Fractionation data acquired in ddaPASEF was analyzed with AlphaPept to obtain a deep proteome coverage of each tumor bulk sample. The resulting output table was then imported into Perseus,^72^ and filtered for protein groups with 70% of quantitative values present ‘in at least one group’ (C, HDA, NMN). DIA-NN output tables were similarly processed in Perseus. Before statistical testing, missing values were imputed based on a normal distribution (width = 0.3; downshift = 1.5). To correct for multiple hypothesis testing in pairwise proteomic comparisons (two-sided unpaired t test), we applied a permutation-based false discovery rate (FDR) of either 5% or 1%, as specified in the figure legends. We corrected a multi-sample ANOVA for a 1% false discovery rate (FDR). Gene Set Enrichment Analysis (GSEA) was done in Python 3.9.7 (https://github.com/zqfang/GSEApy, v1.0.4). For visualization, we used the Python libraries NumPy (v1.20.3), Pandas (v1.3.4), Matplotlib (v3.4.3), and Seaborn (v0.12.2). Gene ontology (GO) term enrichment analysis was performed online utilizing the ShinyGo application (http://bioinformatics.sdstate.edu/go/, v0.77).

## Supplemental information

Document S1. Figures S1–S4, Tables S1, and S2

Data S1. Significantly enriched proteins from pairwise proteomic comparisons and ANOVA, related to Figure 4
