# Supplementary material for: Spatial characterization and stratification of colorectal adenomas by deep visual proteomics
Source: iScience. 2024 Jul 31;27(9):110620. doi: 10.1016/j.isci.2024.110620 (PMC11381895; doi:10.1016/j.isci.2024.110620)
Supplement: Document S1. Figures S1–S4, Tables S1, and S2 [file mmc1.pdf]

iScience, Volume *27*

Supplemental information

Spatial characterization and stratification

# of colorectal adenomas by deep visual proteomics

Sonja Kabatnik, Frederik Post, Lylia Drici, Annette Snejbjerg Bartels, Maximilian T. Strauss, Xiang Zheng, Gunvor I. Madsen, Andreas Mund, Florian A. Rosenberger, José Moreira, and Matthias Mann

Supplemental information

Spatial characterization and stratification of colorectal adenomas by Deep Visual Proteomics

Sonja Kabatnik, Frederik Post, Lylia Drici, Annette Snejbjerg Bartels, Maximilian T Strauss, Xiang Zheng, Gunvor Iben Madsen, Andreas Mund, Florian A Rosenberger, José MA Moreira, and Matthias Mann

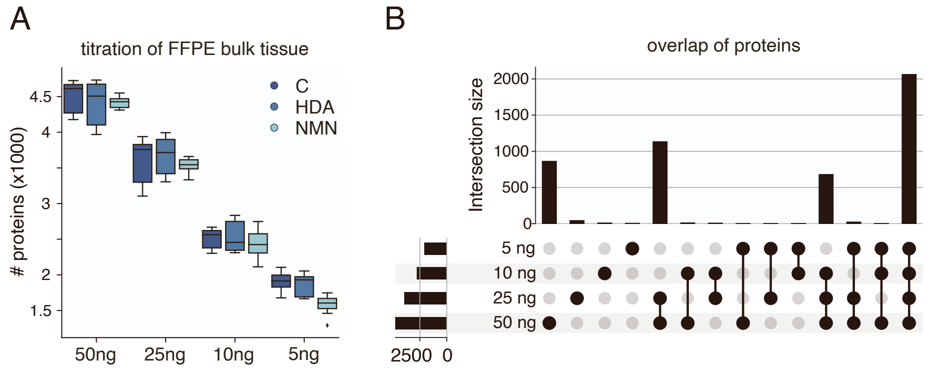

Supplemental Figure S1 Biological and comparative analysis of DIA acquired data from bulk CRA FFPE samples, related to Figure 2.

(A) Number of unique proteins at decreasing FFPE bulk lysate injections (50ng, 25 ng, 10 ng and 5 ng), measured in DIA and quantified by library-based DIA-NN.

(B) Overlap of proteins across different injection amounts.

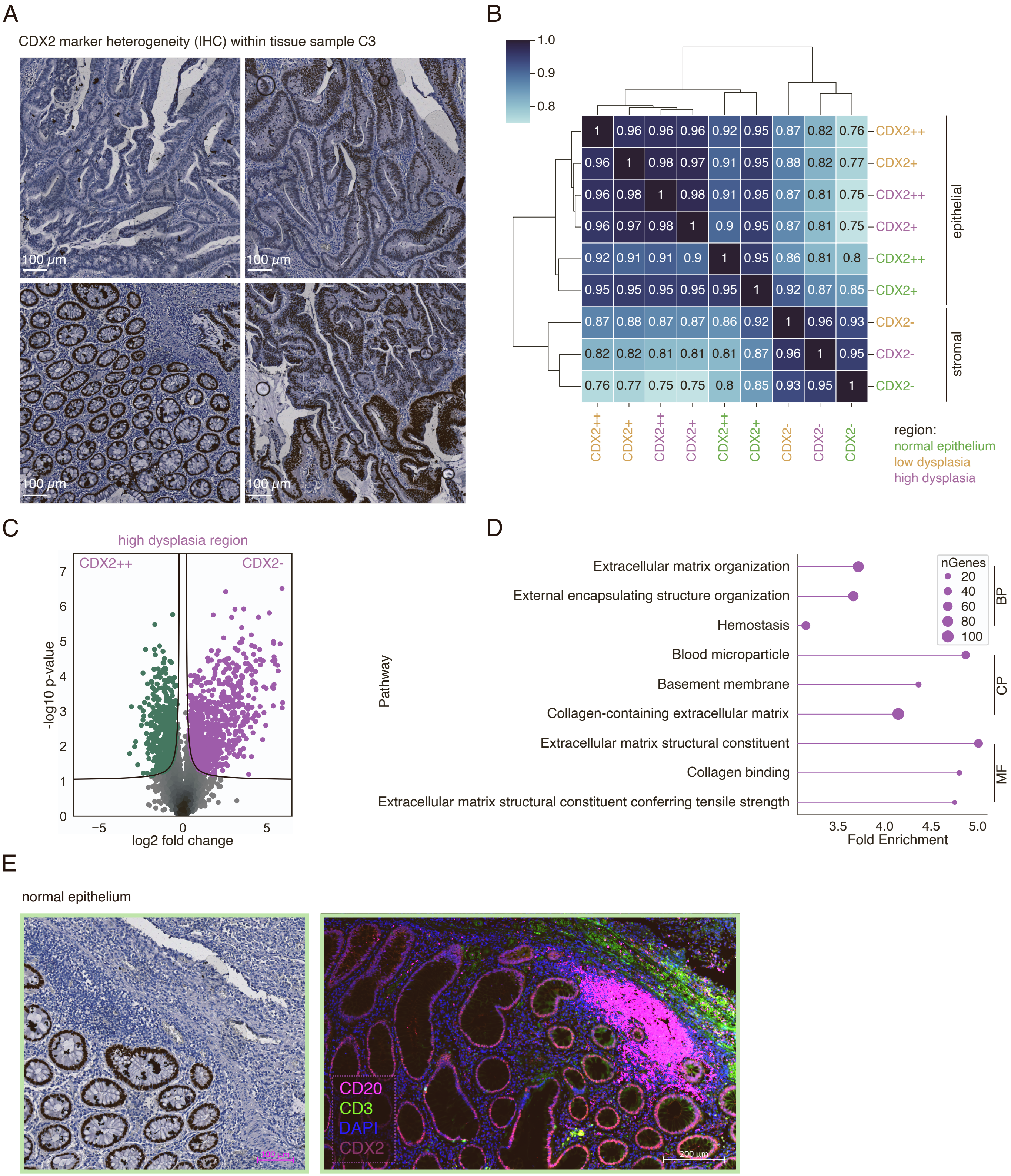
A
CDX2 marker heterogeneity (IHC) within tissue sample C3
100 μm
100 μm
100 μm
100 μm
B
1.0
0.9
0.8
1 0.96 0.96 0.96 0.92 0.95 0.87 0.82 0.76 CDX2++
0.96 1 0.98 0.97 0.91 0.95 0.88 0.82 0.77 CDX2+
0.96 0.98 1 0.98 0.91 0.95 0.87 0.81 0.75 CDX2++
0.96 0.97 0.98 1 0.9 0.95 0.87 0.81 0.75 CDX2+
0.92 0.91 0.91 0.9 1 0.95 0.86 0.81 0.8 CDX2++
0.95 0.95 0.95 0.95 0.95 1 0.92 0.87 0.85 CDX2+
0.87 0.88 0.87 0.87 0.86 0.92 1 0.96 0.93 CDX2-
0.82 0.82 0.81 0.81 0.81 0.87 0.96 1 0.95 CDX2-
0.76 0.77 0.75 0.75 0.8 0.85 0.93 0.95 1 CDX2-
epithelial
stromal
CDX2++ CDX2+ CDX2++ CDX2+ CDX2++ CDX2+ CDX2- CDX2- CDX2-
region:
normal epithelium
low dysplasia
high dysplasia
C
high dysplasia region
CDX2++
CDX2-
-log10 p-value
log2 fold change
D
Extracellular matrix organization
External encapsulating structure organization
Hemostasis
Blood microparticle
Basement membrane
Collagen-containing extracellular matrix
Extracellular matrix structural constituent
Collagen binding
Extracellular matrix structural constituent conferring tensile strength
Pathway
nGenes
20
40
60
80
100
BP
CP
MF
3.5 4.0 4.5 5.0
Fold Enrichment
E
normal epithelium
CD20
CD3
DAPI
CDX2
200 μm

Supplemental Figure S2 Colorectal adenoma heterogeneity, related to Figure 3.

(A) IHC staining against the CDX2 marker protein of one particularly heterogenous colorectal adenoma (CRA) tissue (C3). Scale bar, 100 μm.

(B) Correlation matrix of log2 normalized and imputed intensity values of diaPASEF-acquired low-input DVP samples.

(C) Pairwise proteomic comparison between cell classes CDX2++ (epithelial) and CDX2- (stromal), both laser microdissected from our pre-defined highly dysplastic adenoma region. Significantly enriched proteins are colored and displayed above the black lines (two-sided t-test, permutation-based FDR <0.05, s_0 = 0.1).

(D) GO term enrichment (FDR <0.05) of significantly positive protein hits, showing pathways included in 'Biological Process' (BP), 'Cellular Process' (CP), and 'Molecular Function' (MF).

(E) Area showing normal glandular architecture of CRA C3. Left: IHC against CDX2 and hematoxylin counterstaining. Right: Immunofluorescence staining against B lymphocytes (CD20), T lymphocytes (CD4) a nuclear staining (DAPI), and CDX2.

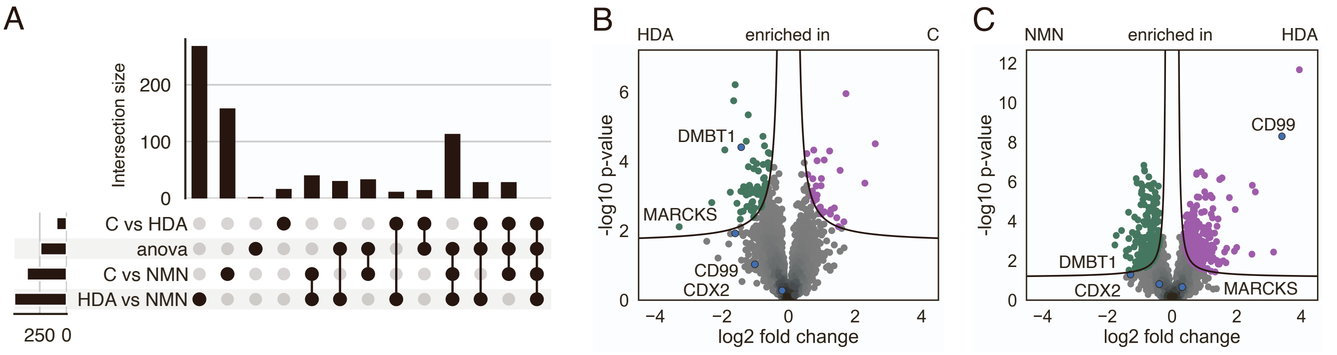

D

C vs NMN

Enriched in C: Protein Group	Gene Name	Enriched in NMN: Protein Group	Gene Name	-log10 p-value: Protein Group	Gene Name
P07148	FABP1	O95620	DUS4L	P26373	RPL13
Q9ULW5	RAB26	Q9BYZ8	REG4	Q92979	EMG1
Q9UGM3-3	DMBT1	Q8NFC6	BOD1L1	P62263	RPS14
Q9NQU5-2	PAK6	A0A087WWM1	MUC1	P05388	RPLP0
P80188	LCN2	Q01995	TAGLN	P30050	RPL12
Q96JA1-2	LRIG1	P14209	CD99	A8MUS3	RPL23A
A6NHN0	OTOL1	Q5T7N2	L1TD1	Q8WXI4-2	ACOT11
A8K7I4	CLCA1	P18065	IGFBP2	P39019	RPS19
Q53HL2	CDCA8	Q6P179	ERAP2	Q9UGM3-3	DMBT1
Q8IYS1	PM20D2	P17661	DES	A8K7Q2	HSPA8
Q9Y2I7	PIKFYVE	O75891-3	ALDH1L1	Q02878	RPL6
A0A0D9SEJ5	FAM120B	D6RF23	RACK1	Q92820	GGH
P12277	CKB	Q92526-3	CCT6B	P62269	RPS18
P16444	DPEP1	U3KQE2	CAPNS1	P12236	SLC25A6
P01859	IGHG2	P61764	STXBP1	P61247	RPS3A
Q9H300	PARL	P30260-2	CDC27	P40429	RPL13A
A0A075B7D8	IGHV3OR15-7	P48163	ME1	P62424	RPL7A
Q6P4A8	PLBD1	P84074	HPCA	Q3YEC7	RABL6
Q9NRD8	DUOX2	C9JZL8	MYADM	Q9BXR0	QTRT1
Q9UPV0	CEP164	Q9HC84	MUC5B	Q14240-2	EIF4A2
Q5BJH7-2	YIF1B	Q9Y4H2	IRS2	P62899	RPL31
P26447	S100A4	P49023-4	PXN	Q86VR2	RETREG3
Q07954	LRP1	Q8N0U8	VKORC1L1	P61313	RPL15
Q96B21	TMEM45B	Q69YN4	VIRMA	D6R9L0	RACK1
P51553	IDH3G	Q9BZF9	UACA	P62753	RPS6
O15120	AGPAT2	Q86TX2	ACOT1	P32969	RPL9
P02786	TFRC	Q7L5N7	LPCAT2	P36578	RPL4
Q9P219	CCDC88C	P08758	ANXA5	P18124	RPL7
Q68CZ6	HAUS3	P02760	AMBP	P46776	RPL27A
P49407	ARRB1	Q96SQ9	CYP2S1	O60506	SYNCRIP

C vs HDA

Enriched in C: Protein Group	Gene Name	Enriched in HDA: Protein Group	Gene Name	-log10 p-value: Protein Group	Gene Name
Q8WWA0	ITLN1	Q7Z333-4	SETX	Q7LG56	RRM2B
A0A087WWM1	MUC1	D6RF23	RACK1	P18065	IGFBP2
P18065	IGFBP2	O43704	SULT1B1	P21266	GSTM3
Q01995	TAGLN	P18827	SDC1	P52758	RIDA
C9JZL8	MYADM	P21266	GSTM3	O00273	DFFA
A0A0B4J259	LYZ	Q7LG56	RRM2B	P10253	GAA
P06731	CEACAM5	Q9NQU5-2	PAK6	Q8WWA0	ITLN1
P09382	LGALS1	P09467	FBP1	P09496-2	CLTA
Q969L2	MAL2	P42765	ACAA2	P29966	MARCKS
Q8NFJ5	GPRC5A	P22570-7	FDXR	O43704	SULT1B1
Q86WV6	TMEM173	P29966	MARCKS	P57735	RAB25
Q8WUY3-2	PRUNE2	P45954	ACADSB	Q86WV6	TMEM173
P08758	ANXA5	Q02318	CYP27A1	P21912	SDHB
Q6EEV4	POLR2M	Q07954	LRP1	Q7Z2K6	ERMP1
O94886	TMEM63A	P19801-2	AOC1	P09874	PARP1
P21333-2	FLNA	P10253	GAA	P08758	ANXA5
Q7Z4F1	LRP10	P35244	RPA3	Q5T6V5	C9orf64
B8ZZT4	VAMP8	O60218	AKR1B10	O75947	ATP5H
P18031	PTPN1	Q5GFL6-2	VWA2	O14936	CASK
Q5T6V5	C9orf64	P52758	RIDA	O94819	KBTBD11
Q9Y223-2	GNE	P02747	C1QC	P29218-3	IMPA1
P00966	ASS1	Q9UHK6-5	AMACR	Q16762	TST
Q15738	NSDHL	Q9BSE5	AGMAT	P02747	C1QC
P57735	RAB25	Q96EL3	MRPL53	P61457	PCBD1
P05783	KRT18	P49748	ACADVL	Q9UIJ7	AK3
Q96G23	CERS2	Q07021	C1QBP	C9JZL8	MYADM
F8WD26	LMO7	P30711	GSTT1	P25815	S100P
Q9Y4H2	IRS2	Q96CM8-2	ACSF2	P37802	TAGLN2
P37802	TAGLN2	Q96GK7	FAHD2A	P17152	TMEM11
P09874	PARP1	Q9H061	TMEM126A	P21333-2	FLNA

HDA vs NMA

Enriched in HDA: Protein Group	Gene Name	Enriched in NMN: Protein Group	Gene Name	-log10 p-value: ProteinGroup	Gene Name
D6RF23	RACK1	P16444	DPEP1	P14209	CD99
P14209	CD99	Q68CZ6	HAUS3	P46776	RPL27A
Q5MY95	ENTPD8	Q9NRD8	DUOX2	P61353	RPL27
U3KQE2	CAPNS1	P26447	S100A4	P18124	RPL7
P10109	FDX1	P07148	FABP1	Q92905	COPS5
Q9Y4C4	MFHAS1	Q16666-2	IFI16	Q6YN16	HSDL2
P01877	IGHA2	Q14657	LAGE3	Q9HBL7	PLGRKT
Q8IVG5	SAMD9L	P02786	TFRC	H3BPR2	NME3
P02461	COL3A1	A0A0D9SEJ5	FAM120B	P04075	ALDOA
Q9Y3Y2-3	CHTOP	P80188	LCN2	G3V2F5	PRMT5
Q9H008	LHPP	Q9HCE7-2	SMURF1	P40429	RPL13A
Q92526-3	CCT6B	Q9UPV0	CEP164	P21266	GSTM3
P48163	ME1	Q9UHX1-6	PUF60	P25205	MCM3
P21695-2	GPD1	Q96EL2	MRPS24	P26373	RPL13
G3V2F5	PRMT5	P05534	HLA-A	P61313	RPL15
P21266	GSTM3	Q6DN03	HIST2H2BC	P15531-2	NME1
Q96T58	SPEN	Q9Y2I7	PIKFYVE	P10109	FDX1
O75891-3	ALDH1L1	Q92820	GGH	P49207	RPL34
P01591	JCHAIN	P40429	RPL13A	Q13310-2	PABPC4
Q96CD0	FBXL8	Q96RQ1	ERGIC2	P50914	RPL14
P62306	SNRPF	P10412	HIST1H1E	P39019	RPS19
P21397	MAOA	P25205	MCM3	C9JNW5	RPL24
H0YD16	TOR1AIP1	F5H124	MAGOHB	P62851	RPS25
Q5T7N2	L1TD1	Q9BTT0-3	ANP32E	P51572-2	BCAP31
P07902-2	GALT	P07195	LDHB	U3KQE2	CAPNS1
Q9BRQ6	CHCHD6	Q8NEW0	SLC30A7	P37802	TAGLN2
Q9BRF8	CPPED1	Q86WV6	TMEM173	P62424	RPL7A
Q9H299	SH3BGRL3	Q99525	HIST1H4G	Q96CM8-2	ACSF2
Q9BWH2	FUNDC2	Q9UIG0-2	BAZ1B	Q14566	MCM6
P11217	PYGM	Q9BWD1	ACAT2	P33993	MCM7

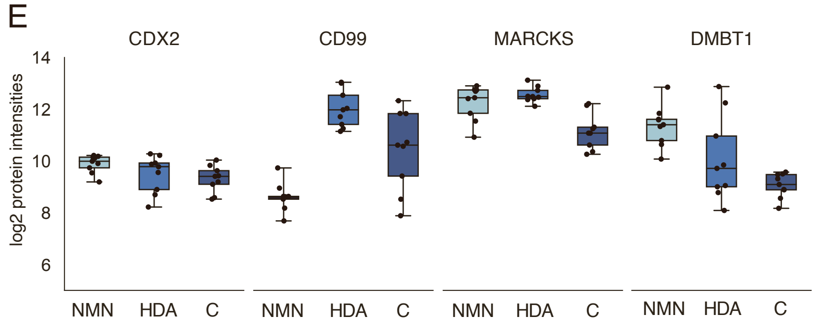

Supplemental Figure S3 Proteomic overview from singly isolated CDX2++ cells from our colorectal adenoma subcohort, related to Figure 4.

(A) Overlap of significantly enriched proteins after pairwise proteomic comparison of CDX2++ cells (two-sided t-test, permutation-based FDR <0.01, $s_0 = 0.1$) across CRA tissues.

(B, C) Pairwise proteomic comparison between group HDA and C, and NMN and HDA (two-sided t-test, permutation-based FDR <0.01, $s_0 = 0.1$).

(D) List of significantly enriched proteins between groups C, HDA and NMN after pairwise proteomic comparison (two-sided t-test, permutation-based FDR <0.01, $s_0 = 0.1$). Top 30 significantly enriched proteins are presented in a descending sequence, ordered according to their -log10 p-value.

(E) Log2-transformed LFQ intensities of marker proteins CD99, DMBT1 and MARCKS.

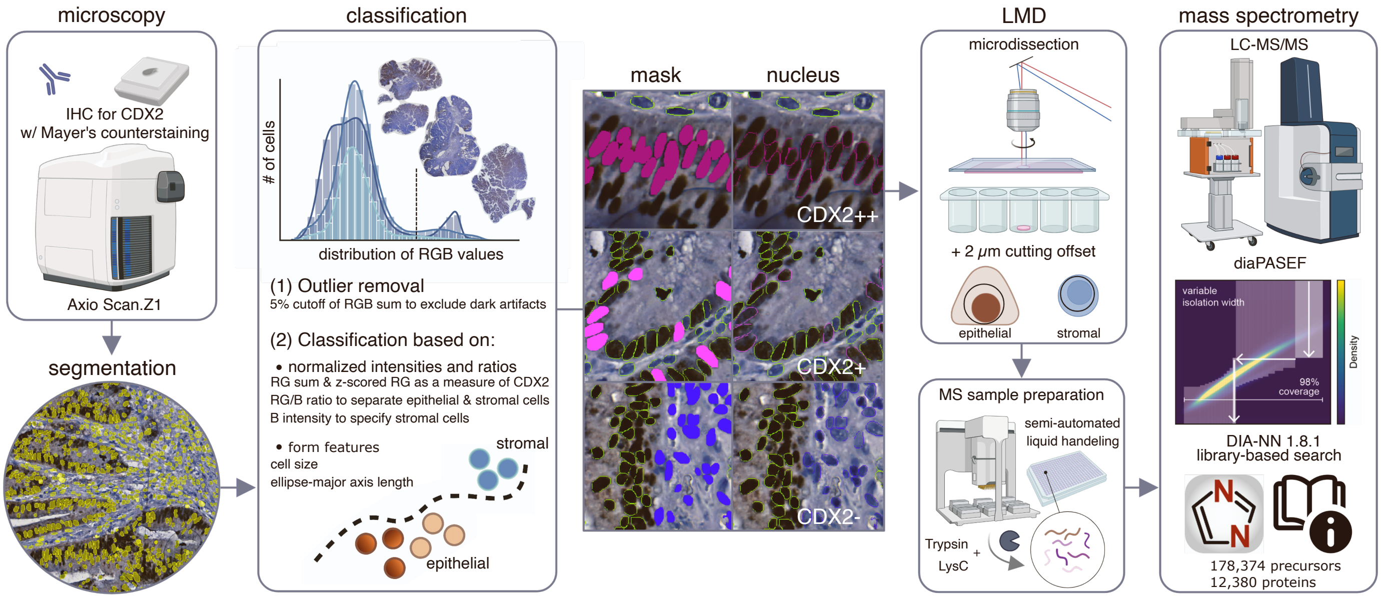

Supplemental Figure S4 Graphical illustration of the DVP workflow, along with the specific criteria used for cell classification, related to the Methods (Cell segmentation and classification). Generated with BioRender.com.

Group	Age range	Sex	Adenoma localization	Recurrence	Relapse type	Tumor type
NMA1	56-60	M	Rectum, distal	-	-	-
NMA2	66-70	M	Rectum, distal	-	-	-
NMA3	76-80	M	Rectum, distal	-	-	-
HDA1	71-75	M	Rectum, distal	1 year	HG adenoma	Tubulovillous
HDA2	71-75	M	Rectum, distal	1 year	HG adenoma	Tubulovillous
HDA3	81-85	M	Rectum, distal	1 year	HG adenoma	Tubulovillous
C1	61-65	M	Rectum, distal	1 year	Adenocarcinoma	Tubulovillous
C2	81-85	M	Rectum, distal	1 year	Adenocarcinoma	Tubulovillous
C3	76-80	M	Colon	1 year	Adenocarcinoma	Tubulovillous

Supplemental Table S1 Colorectal adenoma cohort overview, related to Figure 3.

All nine adenomas were used for cell-type resolved proteomics analysis using the spatial proteomics platform Deep Visual Proteomics.

Group	Age range	Size [mm]	Adenoma localization	Relapse type (time internal [years])
C1	61-65	-	Rectum, distal	Adenocarcinoma (1)
C2	81-85	-	Rectum, distal	Adenocarcinoma (1)
C3	76-80	-	Colon	Adenocarcinoma (1)
C6	71-75	40	-	Adenocarcinoma (1)
C7	61-65	20	Sigmoideum	Adenocarcinoma (1)<br>Low grade neoplasia (3)
C9	71-75	-	Caecum	Adenocarcinoma (1)
HDA1	71-75	-	Rectum, distal	HG adenoma (1)
HDA2	71-75	-	Rectum, distal	HG adenoma (1)
HDA3	81-85	-	Rectum, distal	HG adenoma (1)
HDA7	66-70	-	Colon	HG adenoma
HDA8	76-81	15	Rectum	HG adenoma
HDA9	61-65	Piecemeal	Caecum	HG adenoma
HDA11	66-71	10	Sigmoideum	HG adenoma
NMA1	56-60	-	Rectum, distal	-
NMA2	66-70	-	Rectum, distal	-
NMA3	76-80	-	Rectum, distal	-
NMA10	36-40	-	-	-
NMA11	51-56	40	Rectum	-
NMA12	61-65	35	Rectum	-
NMA13	61-65	45	Sigmoideum	-
NMA14	71-75	Piecemeal	Sigmoideum	-
NMA15	-	-	-	-
NMA16	51-56	-	Caecum	Low grade neoplasia (3)
NMA17	41-46	12	Rectum	-
NMA18	51-56	30	Sigmoideum	Low grade neoplasia (3)

Supplemental Table S2 Colorectal adenoma validation cohort, related to Figure 5.

In addition to the original colorectal adenoma cohort of nine, 16 adenoma FFPE tissues were added.
